# Enhancement of Antibiofilm Activity of Ciprofloxacin against *Staphylococcus aureus* by Administration of Antimicrobial Peptides

**DOI:** 10.3390/antibiotics10101159

**Published:** 2021-09-24

**Authors:** Muhammad Yasir, Debarun Dutta, Mark D. P. Willcox

**Affiliations:** 1 School of Optometry and Vision Science, University of New South Wales, Sydney 2052, Australia; d.dutta@aston.ac.uk (D.D.); m.willcox@unsw.edu.au (M.D.P.W.); 2 Optometry and Vision Science Research Group, Optometry School, University of Aston, Birmingham B4 7ET, UK

**Keywords:** *Staphylococcus aureus*, antibiotic resistance, biofilms, antimicrobial peptides, ciprofloxacin, combined effect

## Abstract

*Staphylococcus aureus* can develop resistance by mutation, transfection or biofilm formation. Resistance was induced in *S. aureus* by growth in sub-inhibitory concentrations of ciprofloxacin for 30 days. The ability of the antimicrobials to disrupt biofilms was determined using crystal violet and live/dead staining. Effects on the cell membranes of biofilm cells were evaluated by measuring release of dyes and ATP, and nucleic acids. None of the strains developed resistance to AMPs while only *S. aureus* ATCC 25923 developed resistance (128 times) to ciprofloxacin after 30 passages. Only peptides reduced biofilms of ciprofloxacin-resistant cells. The antibiofilm effect of melimine with ciprofloxacin was more (27%) than with melimine alone at 1X MIC (*p* < 0.001). Similarly, at 1X MIC the combination of Mel4 and ciprofloxacin produced more (48%) biofilm disruption than Mel4 alone (*p* < 0.001). Combinations of either of the peptides with ciprofloxacin at 2X MIC released ≥ 66 nM ATP, more than either peptide alone (*p* ≤ 0.005). At 2X MIC, only melimine in combination with ciprofloxacin released DNA/RNA which was three times more than that released by melimine alone (*p* = 0.043). These results suggest the potential use of melimine and Mel4 with conventional antibiotics for the treatment of *S. aureus* biofilms.

## 1. Introduction

*Staphylococcus aureus* is a major human pathogen that can cause several recalcitrant infections (deep-seated abscess, osteomyelitis, and endocarditis) due to the acquisition of antibiotic resistance and formation of biofilm on living tissues and medical devices [1,2]. Methicillin-resistant *S. aureus* (MRSA) has been named as a “serious threat” by the Center for Disease Control and Prevention [3,4]. Approximately 11,000 people die each year from a MRSA-related infection in the United States alone [5,6]. So far, there are limited reports on antimicrobial compounds that are able to control biofilm-associated infections caused by *S. aureus* [7].

Various strategies such as physical removal of materials colonized with bacteria or delivery of high doses of antibiotics at the site of infections have been used to treat biofilm-associated infection [8]. However, due to poor penetration of antibiotics through the extracellular polysaccharide matrix of biofilms and survival of biofilm-embedded cells, even the use of high levels of antibiotics can result in low cure rates for infections [9]. Moreover, high doses of antibiotics may cause cytotoxicity to human cells. Therefore, combinations of different antimicrobials may be required [10].

Several antimicrobial peptides (AMPs) are known to have strong antibiofilm activity against bacterial biofilms [11,12,13]. They can prevent bacterial attachment to surfaces (a first step toward biofilm formation) and destroy already developed biofilms by causing detachment or killing of biofilm-embedded cells [11,13,14]. They can also enhance the activity of antibiotics against biofilms when used in combination [13,15,16,17]. These combined treatments may become an important part of treating biofilm-related infections, such as chronic wounds or biomaterial-associated infections caused by *S. aureus* [18]. In combination treatments, one mode of action that has been proposed is that the antibiotics bind to teichoic acids of staphylococcal cell wall which reduces the interaction with AMPs and facilitates their interaction with bacterial membranes. In this way, AMPs act on the cell membranes and antibiotics target cell wall and/or inhibit biosynthesis of nucleic acids and proteins [19,20].

Melimine (TLISWIKNKRKQRPRVSRRRRRRGGRRRR) and Mel4 (KNKRKRRRRRRGGRRRR) are cationic AMPs which have a wide spectrum of activity targeting clinical isolates of Gram-negative and Gram-positive bacteria (including MRSA and multidrug-resistant *P. aeruginosa*), fungi and protozoa such as *Acanthamoeba* [21,22]. Both AMPs are non-cytotoxic at well above active concentrations [21,22]. Melimine causes hemolysis of horse red blood cells at concentrations 15 times higher than its minimum inhibitory concentration (MIC) [23] while Mel4 causes < 5% hemolysis even at concentrations 17 times higher than its MIC [23]. Melimine and Mel4 can synergize with ciprofloxacin against planktonic as well as biofilm forms of *P. aeruginosa* [24]. Ciprofloxacin is a broad-spectrum antibiotic, active against both Gram-positive and Gram-negative bacteria. Ciprofloxacin kills bacteria by binding to bacterial enzymes DNA gyrase and topoisomerase IV. After binding, the enzyme undergoes conformational changes and breaks the DNA, and ciprofloxacin prevents religation of the broken DNA which ultimately stops DNA replication [25]. Both AMPs in combination with ciprofloxacin destroy *P. aeruginosa* biofilms at concentrations lower than their MICs [13]. Both AMPs act on the cell membranes of planktonic cells of *P. aeruginosa* and this results in release of cellular contents [13]. However, it is not known whether peptides alone or in combination with antibiotics are active against *S. aureus* biofilms or can act in a similar way as they do to *P. aeruginosa* biofilms. The current study investigates the interaction of AMPs melimine or Me4 alone or in combination with ciprofloxacin against *S. aureus* biofilm in conjunction with their mode of activity.

## 2. Results

### 2.1. Minimal Inhibitory Concentration and Minimal Bactericidal Concentration

Table 1 represents the MICs and MBCs values of both the peptides and ciprofloxacin. Melimine and Mel4 had the lowest MICs of 62.5 µg/mL and 125 µg/mL, respectively, against *S. aureus* ATCC 6538. For all other strains, there were slightly higher MICs, 125 µg/mL for melimine and 250 µg/mL for Mel4, except for *S. aureus* ATCC 25923 for which Mel4 had the highest MIC value of 500 µg/mL (Table 1). Ciprofloxacin had similar MICs (0.5 µg/mL) and MBCs (1 µg/mL) against all the tested strains except for *S. aureus* ATCC 6538 for which ciprofloxacin had the same MIC and MBC values of 0.5 µg/mL (Table 1).

### 2.2. Development of Resistance to AMPs and Ciprofloxacin

**Figure 1:**
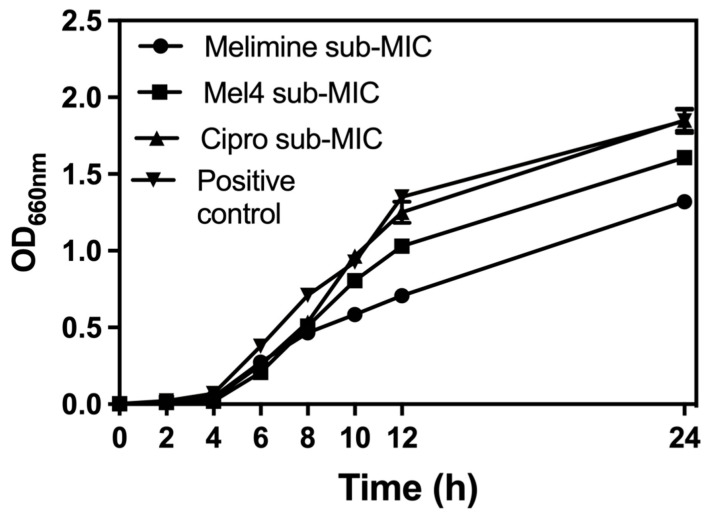
Growth curves for *S. aureus* ATCC 25923 at sub-MIC of the antimicrobial peptides (AMPs) melimine and Mel4 or ciprofloxacin (Cipro). Melimine and Mel4 reduced the overall bacterial growth over 24 h of experiments while ciprofloxacin and the positive control (without any antimicrobial) had similar growth characteristics after 24 h experiment.

The growth curves of *S. aureus* ATCC 25923 at sub-MICs of melimine, Mel4 or ciprofloxacin over 24 h are presented in Figure 1. The growth of *S. aureus* ATCC 25923 at its sub-MIC for ciprofloxacin was similar to growth without the antimicrobial. Melimine and Mel4 affected the growth rate of *S. aureus* after 6 h. Exposure to melimine resulted in slightly less growth than exposure to Mel4 over 24 h.

**Figure 2:**
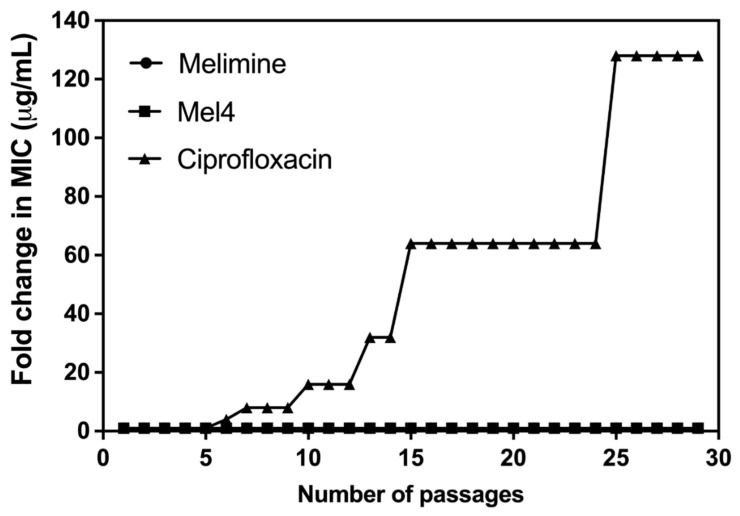
Increase in MIC values of ciprofloxacin, melimine or Mel4 against *S. aureus* ATCC 25923 after exposing bacteria at their sub-MIC for 30 consecutive days. The MIC values of melimine and Mel4 did not change over time and overlap at the bottom of the figure.

Of all the tested strains, only *S. aureus* ATCC 25923 was able to develop resistance to ciprofloxacin. Changes in MICs of *S. aureus* ATCC 25923 after exposure to sub-MICs of melimine, Mel4 or ciprofloxacin over 30 days are presented in Figure 2. The MICs of melimine and Mel4 did not change over time, suggesting a limited potential of resistance development to these peptides. Compared to the peptides, there was rapid development of resistance to ciprofloxacin. Resistance developed to ciprofloxacin after 7 days of serial passage with an initial 4-fold increase in MIC. The MIC increased 64-fold after 15 passages and 128-fold by 30 passages (Figure 2).

### 2.3. Inhibition of Biofilm Formation by AMPs and Ciprofloxacin Alone or in Combination

**Figure 3:**
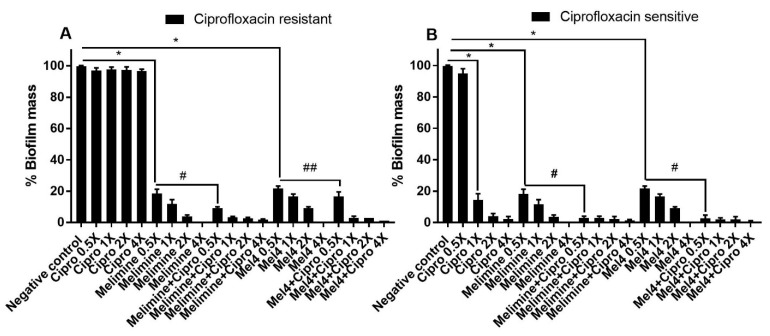
Inhibition of biofilm formation of *S. aureus* ATCC 25923. Biofilm formation of the ciprofloxacin-resistant (**A**) or sensitive (**B**) cells of *S. aureus* ATCC was inhibited by various concentrations of melimine, Mel4 and ciprofloxacin alone or in combination. The strain was made resistant to ciprofloxacin by sub-passage for 30 days at a sub-MIC concentration. * represent significant (*p* < 0.001) decreases compared to the negative control (bacteria grown in the absence of antibiotics). ^#^ indicates significant (*p* < 0.001) decrease for the combinations compared to melimine or Mel4 alone while ^##^ indicates *p* = 0.036 compared to Mel4 alone. Means (±SD) of three independent repeats in triplicate. Negative control = bacteria grown in the absence of antimicrobials, Cipro = ciprofloxacin.

Ciprofloxacin did not inhibit the biofilm formation of the ciprofloxacin-resistant cells of *S. aureus* ATCC 25923 at any concentration tested (*p* > 0.999; Figure 3A). Melimine and Mel4 inhibited biofilm formation at 0.5X MIC by 82% and 78%, respectively, compared to the negative control (*p* < 0.001). There was similar biofilm inhibition with both the AMPs at 0.5X MIC (*p* > 0.999). However, combined use of melimine with ciprofloxacin at 0.5X MICs resulted in 91% inhibition of biofilm, and this inhibition was significantly higher (*p* < 0.001) than the 82% produced by melimine alone at 0.5X MICs (Figure 3A). Similarly, Mel4 and ciprofloxacin in combination at 0.5X MIC produced 83% inhibition of biofilm which was significantly higher (*p* = 0.036) than the 78% produced by Mel4 alone (Figure 3A). There was no significant difference in biofilm inhibition between melimine and ciprofloxacin, and Mel4 and ciprofloxacin combinations at 0.5X MIC (*p* > 0.999).

The biofilms produced by the ciprofloxacin-sensitive cells of ATCC 25923 were inhibited by ≥86% by ciprofloxacin at ≥1X MIC (*p* < 0.001; Figure 3B). Melimine or Mel4 were active at 0.5X MICs and produced 82% and 78% biofilms inhibition compared to negative control, respectively (*p* < 0.001). The combinations of melimine or Mel4 with ciprofloxacin at 0.5X MIC produced reductions that were significantly higher (97%) than those used alone at 0.5X (*p* < 0.001). The combinations of either AMP with ciprofloxacin inhibited the same amount of biofilm at 0.5X MICs (*p* > 0.999; Figure 3B).

### 2.4. Disruption of Pre-Formed Biofilms by AMPs and Ciprofloxacin Alone or in Combination

In comparison to the effect of the AMPs or the combination of AMPs with ciprofloxacin on preventing the production of biofilms, all were less active in reducing pre-formed biofilms. For melimine or Mel4 at 0.5X to 2X MIC, pre-formed biofilms of either the ciprofloxacin-resistant or sensitive cells were 4–6 times more resistant than the biofilms formed in the presence of melimine.

**Figure 4:**
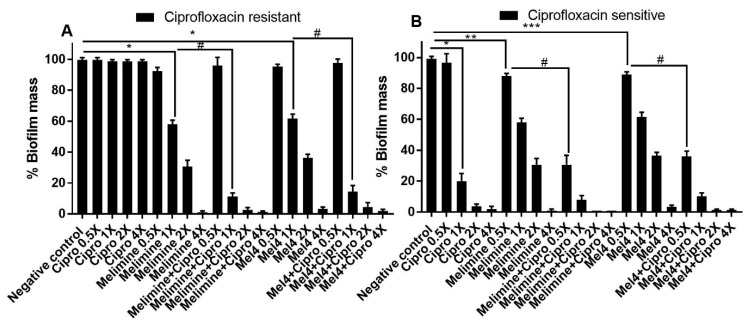
Disruption of pre-established biofilm of *S. aureus* ATCC. Biofilms of the ciprofloxacin-resistant (**A**) and sensitive (**B**) cells of *P. aeruginosa* ATCC 27853 were disrupted at various concentrations by melimine, Mel4 and ciprofloxacin alone or in combination. * represents significant (*p* < 0.001), ** indicates significant (*p* = 0.005), *** indicates significant (*p* = 0.022) decrease compared to the negative control (biofilm treated with buffer). ^#^ indicates significant (*p* < 0.001) decrease for the combinations compared to melimine or Mel4 alone. Error bars represent means (±SD) of three independent repeats in triplicate. Negative control = bacteria grown in the absence of antimicrobials. Cipro = ciprofloxacin.

The ability of AMPs and ciprofloxacin alone or in combination to disrupt pre-formed (24 h) biofilms of ciprofloxacin-resistant and sensitive isolates of *S. aureus* ATCC 25923 is presented in Figure 4. Ciprofloxacin did not reduce pre-formed biofilms of the ciprofloxacin-resistant isolate of *S. aureus* ATCC 25923 at any of the concentrations tested (*p* > 0.999; Figure 4A). Both AMPs reduced the amount of pre-formed biofilms in a concentration-dependent manner except at 0.5X MIC. Melimine produced 42%, 69% and 100% while Mel4 disrupted 38%, 64% and 97% at 1X, 2X and 4X MICs compared to negative control, respectively (*p* < 0.001; Figure 4A). Disruption of biofilm by melimine and Mel4 was similar at their corresponding MICs (*p* > 0.999). The combination of melimine and ciprofloxacin resulted in 69% biofilm disruption and the combination of Mel4 and ciprofloxacin resulted in 86% biofilm disruption at their corresponding 1X MIC compared to negative control (*p* < 0.001). The combined treatment of either AMP with ciprofloxacin at 1X MIC resulted in similar biofilm disruption (*p* > 0.999).

Pre-formed biofilms of the ciprofloxacin-sensitive strain of *S. aureus* ATCC 25923 were susceptible to the action of ciprofloxacin at 1X MIC or higher concentrations. Ciprofloxacin disrupted pre-formed biofilms in a dose-dependent manner by producing 86%, 96% and 100% disruption of biofilms at 1X, 2X and 4X MICs, respectively, compared to control (*p* < 0.001; Figure 4B). Melimine disrupted 11% (*p* = 0.005) and Mel4 disrupted 10% (*p* = 0.022) of pre-formed biofilms compared to negative control at 0.5X MIC. At 1X MIC, melimine eradicated 41% of biofilm while Mel4 eradicated 37% of biofilm compared to buffer-treated negative controls (Figure 4B; *p* < 0.001). Interestingly, when AMPs were used in combination with ciprofloxacin, these combinations resulted in higher pre-formed biofilm disruption at concentrations lower than their MICs. The combination of melimine with ciprofloxacin at 0.5X MIC produced significantly higher (68%) biofilm disruption than when melimine (11%) was used alone at 0.5X (Figure 4B; *p* < 0.001). Similarly, the combination of Mel4 with ciprofloxacin at 0.5X MIC produced significantly higher (63%) biofilm disruption than when Mel4 (10%) was used alone at 0.5X (Figure 4B; *p* < 0.001). The combined treatment of either AMP with ciprofloxacin at 0.5X MIC resulted in similar biofilm disruption (*p* > 0.999). Similarly, at 1X MIC the combination of melimine with ciprofloxacin disrupted more highly (91%) than by melimine alone (41%) and Mel4 and ciprofloxacin disrupted more (89%) than by Mel4 alone (37%; *p* < 0.001). The combined antibiofilm effect of either peptide with ciprofloxacin was similar at 1X MIC (*p* > 0.999).

### 2.5. Visualization of Biofilms

**Figure 5:**
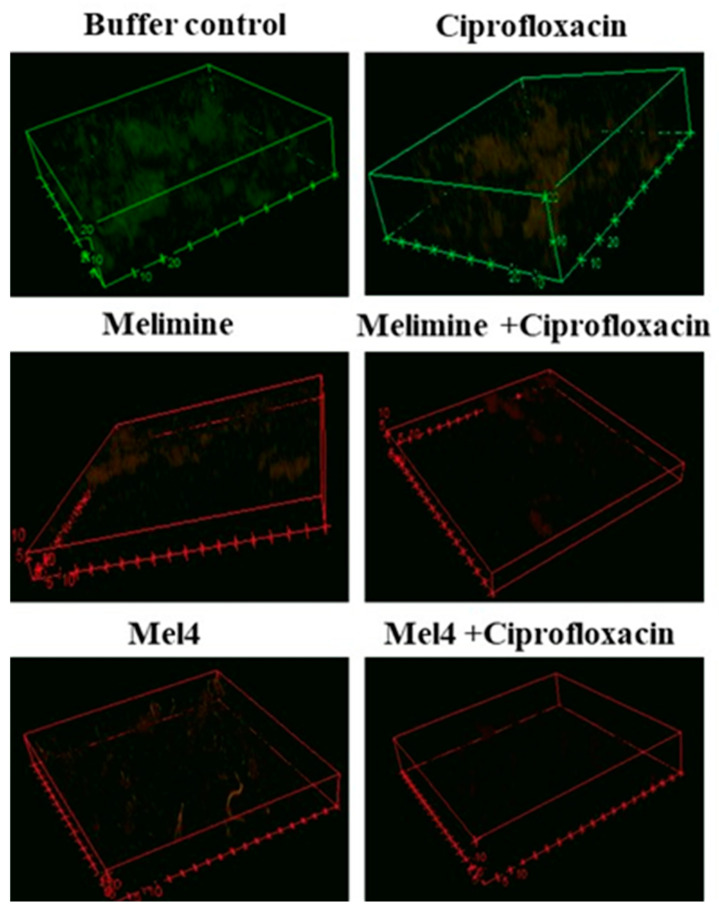
Representative confocal laser scanning microscopy images of biofilms of the ciprofloxacin resistant isolates of *S. aureus* ATCC 25923 after treatment with AMPs and ciprofloxacin alone or in combination. The antibiofilm effects were evaluated at 4X the MIC of all antimicrobials after incubation for 24 h. The biofilms of *S. aureus* were stained with SYTO-9 (excited at 488, green live cells) and propidium iodide (excited at 514 mm, red dead cells). The cells exposed to ciprofloxacin alone when excited at 514 nm had a reddish color indicating some of the cells had taken up the propidium iodide.

Biofilms of the ciprofloxacin-resistant cells treated with buffer (HEPES) or ciprofloxacin alone had an overall dimension of 90 µm by 90 µm by 21 µm and the cells were mainly green, indicating that they were alive (Figure 5). Biofilms treated with melimine or Mel4 at 4X their MICs had less biofilm mass with dimensions of 43 µm by 43 µm by 6 µm and the cells were mainly stained red indicating many dead cells. No biofilms could be seen for the melimine and ciprofloxacin or Mel4 and ciprofloxacin combinations at 4X MICs (Figure 5).

### 2.6. Mechanistic Studies

#### 2.6.1. Cell Membrane Depolarization

**Figure 6:**
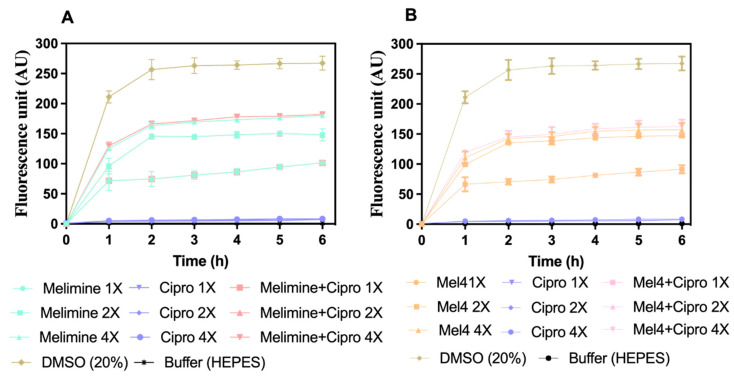
Cell membrane depolarization of pre-formed (24 h) biofilm cells. Cell membrane depolarization of *S. aureus* ATCC 25923 (became resistant to ciprofloxacin after 30 days of serial passages at sub-MIC) (**A**) by melimine and ciprofloxacin alone or in combination, and (**B**) by Mel4 and ciprofloxacin alone or in combination against pre-formed (24 h) biofilms. Error bars are means (±SD) of three independent repeats in triplicate. Cipro = ciprofloxacin, DMSO = dimethyl sulfoxide.

Melimine and Mel4 depolarized the cell membrane of *S. aureus* in biofilms in a concentration- and time-dependent manner (Figure 6A,B). Both peptides depolarized the cell membrane of biofilm cells within 1 h of incubation at 1X, 2X and 4X MICs. The fluorescence intensity produced as a result of the release of the DiSC3 (5) dye was higher at 4X than at 2X and 1X MIC for both melimine and Mel4 (*p* ≤ 0.004). The rate of release of the dye increased up to 2 h and became constant thereafter for all concentrations. There was no difference in release of dye between melimine and Mel4 at their corresponding MICs (*p* ≥ 0.999). Ciprofloxacin did not depolarize the cell membrane at any of the concentrations tested over the entire 6 h of the experiment. The combined membrane depolarizing effect of melimine or Mel4 with ciprofloxacin was almost exactly equivalent to the individual effects of melimine or Mel4 at their corresponding 1X, 2X, and 4X MICs (*p* > 0.937; Figure 6A,B). There was no difference between the combinations at 1X and 2X MICs (*p* > 0.999). However, at 4X MIC, the melimine and ciprofloxacin combination caused higher membrane depolarization than the Mel4 and ciprofloxacin combination after 2 h of incubation (*p* = 0.005). The positive control (DMSO 20%) gave maximum fluorescence at 2 h which became constant following this time point.

#### 2.6.2. Release of Cellular Contents

**Figure 7:**
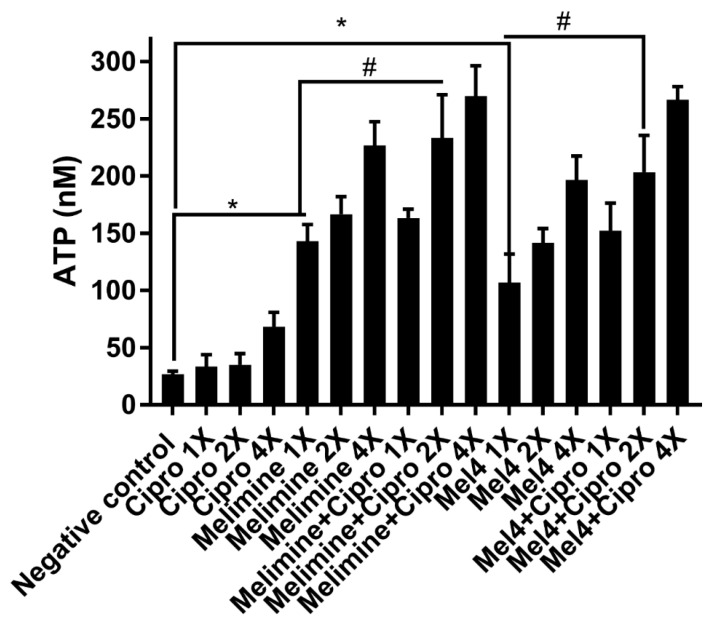
Leakage of ATP from pre-formed biofilm cells of *S. aureus* ATCC 25923. Leakage of ATP following treatment for 3 h with either of the two peptides and ciprofloxacin alone or in combination. The strain was made resistant to ciprofloxacin by passage for 30 days at a sub-MIC. * represents significant (*p* < 0.001) increases in the amount of extracellular at inhibitory concentrations of peptides ATP compared to the negative control. # represents significant (*p* < 0.001) increase in the release of ATP of the combination of melimine or Mel4 with ciprofloxacin compared to melimine or Mel4 alone.

Incubation of the AMPs with pre-formed biofilms of *S. aureus* ATCC 25923 released a substantial amount of ATP in a concentration-dependent manner (Figure 7). Melimine at 1X, 2X and 4X MIC induced leakage of 143 ± 15 nM, 167 ± 15 nM and 227 ± 21 nM ATP, respectively, compared to buffer-treated negative controls (*p* < 0.001). Mel4 at 1X, 2X and 4X MICs released 107 ± 25 nM, 142 ± 13 nM and 197 ± 21 nM extracellular ATP, respectively, compared to negative control (*p* ≤ 0.003). The amount of ATP released by melimine and Mel4 at their corresponding MICs was similar (*p* ≥ 0.999). The addition of ciprofloxacin alone to pre-formed biofilms did not result in the significant release of extracellular ATP at any of the concentrations tested (*p* > 0.999; Figure 7). However, the combination of melimine or Mel4 with ciprofloxacin resulted in the release of higher amounts of ATP than the AMPs alone. At 2X MIC, the melimine and ciprofloxacin combination released significantly higher amounts of ATP (233 ± 38 nM; *p* = 0.005) than released by melimine alone (167 ± 15 nM). There was similar effect on ATP leakage of the combination at 2X and 4X MICs. The combination of Mel4 and ciprofloxacin at 1X, 2X and 4X concentrations induced leakage of 152 ± 24 nM, 203 ± 32 nM and 267 ± 12 nM ATP, respectively (Figure 7). At 2X MIC, the combination of Mel4 and ciprofloxacin released significantly higher amounts of ATP (*p* = 0.002) than was released by Mel4 alone at 1X MIC. Both the melimine and ciprofloxacin or Mel4 and ciprofloxacin combination had similar effects at their corresponding MICs (*p* > 0.999).

**Figure 8:**
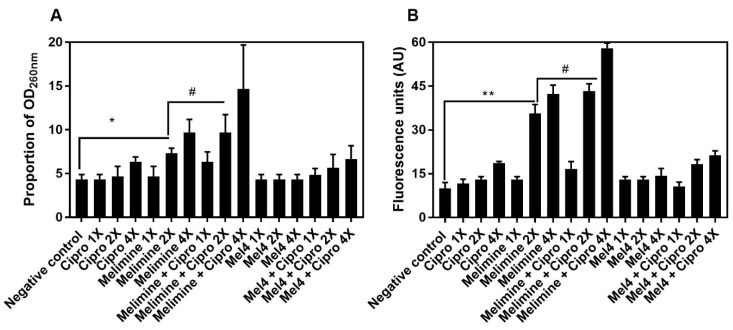
Increase in OD_260nm_ after release of DNA/RNA (**A**) and increase in fluorescence after interaction of Sytox green with released DNA/RNA (**B**) from pre-formed biofilm cells of *S. aureus* ATCC 25923. Leakage of nucleic acid from pre-formed (24 h) biofilms of *S. aureus* ATCC 25923 following treatments for 3 h with either of the two peptides and ciprofloxacin alone or in combination. The strain was made resistant to ciprofloxacin by passage of 30 days at a sub-MIC concentration. * represents significance (*p* = 0.043) and ** indicates (*p* ≤ 0.034) release of nucleic acid compared to the negative control. # represents significant (*p* = 0.022) increase in the release of nucleic acid by the combination of melimine and ciprofloxacin compared to melimine alone.

The release of nucleic acids (260 nm absorbing material) after incubation for 4 h with the antimicrobials from pre-formed biofilms of *S. aureus* ATCC 25923 is shown in Figure 8A. Melimine released a significantly higher amount of DNA/RNA at 2X MIC (7 ± 1 times; *p* = 0.043) and 4X MIC (13 ± 1 times; *p* < 0.001) compared to control. Ciprofloxacin did not cause significant DNA/RNA leakage from the pre-formed biofilms at any concentration tested (*p* > 0.999; Figure 8A). The combination of melimine and ciprofloxacin released 10 ± 2 times (*p* = 0.047) more DNA/RNA compared to negative control at 2X MIC. Melimine and ciprofloxacin in combination released significantly higher (*p* = 0.022; Figure 8A) amounts of DNA/RNA than melimine alone at 2X MIC. The combination of Mel4 and ciprofloxacin did not release significant amounts of DNA/RNA at any concentration tested (*p* ≥ 0.480). Melimine either alone or in combination with ciprofloxacin produced higher fluorescence at 2X and 4X MICs than other concentrations (*p* ≤ 0.034; Figure 8B). Mel4 either alone or in combination with ciprofloxacin did not produce significant fluorescence at any concentration tested (*p* > 0.999; Figure 8B).

## 3. Discussion

Exposure of bacteria to sub-inhibitory concentrations of antimicrobials can result in generation of resistant mutants [26,27]. The current study demonstrated that the AMPs melimine and Mel4 at sub-MICs did not induce resistance in *S. aureus* ATCC 25923. We and others [28,29,30,31] have tested several broad-spectrum antibiotics such as gentamicin (data not shown in the current study) and ciprofloxacin to determine whether strains such as *S. aureus* ATCC 6538, ATCC 25923, 31 and 38 can develop resistance to gentamycin and ciprofloxacin. Resistance to gentamicin or ciprofloxacin was not induced in any strain except *S. aureus* ATCC 25923 which developed resistance against ciprofloxacin. Therefore, ciprofloxacin was selected to determine its activity alone or in combination with antimicrobial peptides against this strain. Biofilms of the resistance cells of *S. aureus* ATCC 25923 could be reduced by treatment with combinations of melimine or Mel4 with ciprofloxacin whilst the biofilm was forming or once it had developed.

*S. aureus* ATCC 25923 developed resistances to ciprofloxacin similar to *P. aeruginosa* ATCC 27853 [13], in a step-wise manner to full resistance (>120X MIC) after 25 days of passage. Resistance to ciprofloxacin in *S. aureus* can occur due to mutations in *grlA/grlB* and *gyrA/gyrB* genes, which encode the subunits of topoisomerase IV and DNA gyrase, respectively [32,33], or over expression of the membrane-associated protein NorA efflux pump which leads to increased transport of ciprofloxacin out of the bacterial cell [34]. Changes in these genes may occur randomly during exposure to ciprofloxacin and this may be why the resistance occurs sporadically during exposure to the antibiotic. In contrast to *S. aureus* ATCC 25923, all other *S. aureus* strains (31, 38 and ATCC 6538) did not mutate and develop resistance against ciprofloxacin. None of the *S. aureus* strains was able to develop resistance against melimine and Mel4. The inability of *S. aureus* to develop resistance against melimine and Mel4 may be due to the rapid killing kinetics of these peptides and action on cell membranes [23]. Bacteria appear to rarely gain resistance to AMPs that target bacterial membranes [23,35]. However, like other Gram-positive bacteria, *S. aureus* can develop resistance to AMPs by reducing the negative charge on teichoic acid and production of proteases that fragment AMPs [36,37], but these mechanisms appear not to have been activated during growth in sub-MICs of melimine or Mel4.

Another mechanism whereby bacteria can protect themselves from the action of antimicrobials is formation of biofilms [38]. Melimine and Mel4 prevented biofilm formation of *S. aureus* at a concentration lower than their MICs. A similar effect has been shown with the cathelicidin-derived peptide NA-CATH:ATRA1-ATRA1 against *S. aureus* biofilm [39]. The AMPs esculentin-3, Tet-213 and 1010 peptides prevent biofilm formation [40,41] by stimulating twitching motility, influencing quorum sensing or degrading signaling molecules such as ppGpp which lead to changes in the expression of genes related to biofilm assembly [42,43,44].

Melimine and Mel4 killed biofilm cells and dispersed pre-formed biofilms. Similarly, AMPs such as LL37, DL-K6L9, Seg5L, Seg5D, Seg6L, and Seg6D killed the biofilm cells and reduced the biofilm mass by dispersing the biofilm matrix [45,46]. Both our AMPs followed a similar mechanism, as treating biofilms of ciprofloxacin-resistant cells with either AMP resulted in a high proportion of PI positive (stained red = dead cells) with a reduced biofilm mass compared to buffer-treated negative controls. Disruption of pre-formed biofilm by these two AMPs was similar to disruption of pre-formed biofilm of *P. aeruginosa* [13]. Like the case with *P. aeruginosa*, the anti-biofilm effects of melimine and Mel4 against *S. aureus* were similar to their mode of action on *S. aureus* cells in suspension [23]; this is depolarization of membranes and release of intracellular contents.

However, the speed of the effects of melimine and Mel4 was decreased compared to their effects on planktonic cells [13], which may be due to the complex structure of *S. aureus* biofilms hindering the antimicrobial action of AMPs. Membrane depolarization of biofilm cells caused by melimine and Mel4 was slower and happened after one hour compared to only 30 s against planktonic bacteria [23]. Similarly, membrane depolarization of *S. aureus* cells in biofilms occurred after 1 h with the AMPs nisin A and lacticin Q [47]. The time required to depolarize the membrane of *S. aureus* biofilm cells was similar to *P. aeruginosa* biofilm cells [13]. Slower membrane depolarization of biofilm cells compared to planktonic bacteria might be due to higher viscosity of biofilm which can affect the penetration of AMPs in biofilm [47,48,49]. Moreover, negatively charged polymers of biofilms may interact with the positively charged AMPs and limit penetration and diffusion of AMPs in biofilm matrix.

Both AMPs killed the biofilm cells by damaging the membranes followed by leakage of cellular ATP. Leakage of ATP from biofilm cells was slower and occurred after 3 h compared to after 2 min from planktonic bacteria [23]. As discussed above, this change in timing of events may be due to the charge of biofilm polymers or viscosity within biofilms. Higher concentrations of AMPs above their MICs may disrupt the membrane of biofilm cells to a greater extent and start to release larger molecules [48,50,51,52]. Melimine released DNA/RNA from biofilm cells at 4X MIC. On the other hand, Mel4 alone or in combination with ciprofloxacin did not result in release of DNA/RNA even at 4X its MIC. The mechanism of action of Mel4 against biofilm cells seems to be similar to planktonic cells which are independent of the release of DNA/RNA [23].

**Figure 9:**
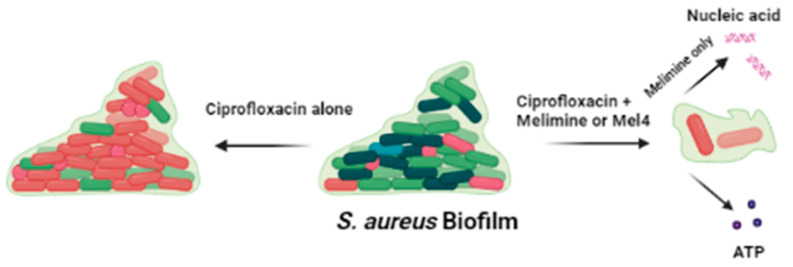
Effect of ciprofloxacin and peptides on the pre-formed biofilm of *S. aureus.* Ciprofloxacin alone did not disrupt the biofilm while when in combination with melimine or Mel4 it destroys the biofilm matrix following release of DNA/RNA (with melimine only) and ATP from biofilm cells.

The combination of AMPs and ciprofloxacin inhibited greater biofilm formation at 0.5X than alone, suggesting that both the peptides may have additive or synergistic effects against *S. aureus*. The AMPs indolicidin, cecropin (1–7) and nisin in combination with ciprofloxacin inhibited the *S. aureus* biofilm at concentrations lower than their MICs [38]. The fractional inhibitory concentrations of these AMPs with ciprofloxacin were above synergistic levels, showing additive effects instead, against planktonic *S. aureus* [24]. The combination of AMPs with ciprofloxacin resulted in more biofilm disruption at 1X MIC than alone. These results coincide with the previous study which reported that the AMPs indolicidin, cecropin (1–7)–melittin A (2–9) and nisin in combination with teicoplanin or ciprofloxacin disrupted the biofilm of methicillin-resistant *S. aureus* at 1X MIC [53]. Smaller differences in biofilms inhibition/disruption may be due to sensitivity of the strain towards antibiotics, maturation of biofilms and concentration of antimicrobials used. Several peptides in combination with antibiotics have been tested against biofilms formed for 2 h to 4 h, at concentrations 2–4 times lower than their MICs. Table 2 compares these combinations with melimine or Mel4 with ciprofloxacin tested at their 0.5X MICs against biofilms formed for 24 h in the present study. The slightly higher effects of the combination of Citropin1.1 + Minocycline [54] or LL37 + Teicoplanin [20] may be due to the fact the biofilms were only produced for 4 h, whereas the current study used biofilms formed over 24 h and these longer times might produce more robust biofilms. The effect of both the peptides with ciprofloxacin against *S. aureus* biofilm is summarized in Figure 9. The ability of the AMP-ciprofloxacin combinations to disrupt greater amounts of pre-formed biofilms might be related to AMPs’ facilitating higher intracellular uptake of ciprofloxacin [55]. The AMPs WR12, SAAP-148, SAAP-276 and TC84 allowed greater cellular uptake of ciprofloxacin and teicoplanin by permeabilizing the cell membrane of *S. aureus* in biofilms [20,55]. Another possible mechanism of AMP-antibiotic combinations is disrupting the biofilm matrix to allow AMPs to target the bacterial cells in the biofilm and cause dispersion of cells in the biofilm [56].

## 4. Materials and Methods

### 4.1. Synthesis of Peptides and Bacteria

Melimine and Mel4 were synthesized by conventional solid-phase peptide protocol [58,59] and were procured from the Auspep Peptide Company (Tullamarine, Victoria, Australia). The purity of the peptides was ≥90%. Ciprofloxacin was purchased from Sigma-Aldrich (St Louis, MO, USA). Ciprofloxacin stock solution (5120 µg/mL) in milli Q water was prepared and stored at −30 °C. Bacterial strains such as *S. aureus* 31 (mecA positive) and *S. aureus* 38 (mecA negative; both microbial keratitis isolates) [60] and two reference strains *S. aureus* ATCC 6538 (mecA negative; a human lesion isolate) and *S. aureus* ATCC 25923 were used in the current study.

### 4.2. Minimal Inhibitory Concentration and Minimal Bactericidal Concentration

The minimum inhibitory and minimum bactericidal concentrations of ciprofloxacin were determined using a standard broth microdilution method of the Clinical Laboratory and Standard Institute (CLSI) and a modified version of the CLSI broth microdilution method was used to determine the MIC of antimicrobial peptides [61]. The MIC was set as the lowest concentration that reduced bacterial growth by ≥90% while the MBC was set as the lowest concentration that reduced bacterial growth by >99.99% following enumeration of live bacteria by plate counts compared to bacteria grown in the absence of any antimicrobial.

### 4.3. Growth Curve and Resistance Development at Sub-MIC of Antimicrobials

An aliquot (100 µL) of an overnight culture (1 × 10^6^ CFU/mL) of bacteria was added to an equal volume of each antimicrobial to achieve a sub-MIC (0.5X MIC) in MHB and was incubated at 37 °C with shaking at 120 rpm for 24 h. The turbidity of the bacterial suspensions was determined at OD_660nm_ over time for 24 h. Bacteria grown in wells without antimicrobials served as positive controls for maximum bacterial growth. Serial passages of *S. aureus* ATCC 25923 were performed in the presence of each antimicrobial at 0.5X MIC. After incubation for 18–24 h, cells were repassaged into fresh media containing sub-MICs of the antimicrobials. After every passage, the MIC for each antimicrobial was determined, and a new sub-MIC was adjusted if any increase in MIC was observed. This repassaging lasted for 30 consecutive days. *S. aureus* 31, *S. aureus* 38, *S. aureus* ATCC 6538 and *S. aureus* ATCC 25923 strains were exposed to AMPs and ciprofloxacin at sub-MIC (one-fold below the MIC) for their ability to develop resistance against these antimicrobials. Of all the tested strains, only *S. aureus* ATCC 25923 was able to develop resistance to ciprofloxacin using this method. This strain has been shown to be able to develop resistance to ciprofloxacin previously [28].

### 4.4. Inhibition of Biofilm Formation by AMPs and Ciprofloxacin Alone or in Combination

Inhibition of biofilm formation by AMPs alone or in combination with ciprofloxacin was determined using *S. aureus* 25923 that had been passaged for one day (sensitive cells) or thirty days (resistant cells). First, 100 µL of *S. aureus* (1 × 10^6^ CFU/mL) was dispensed into round-bottom 96-well microtiter plates containing serial dilutions (0.5X to 4X MIC) of melimine, Mel4 or ciprofloxacin. Then plates were incubated at 37 °C with shaking at 120 rpm for 24 h. The combined effect of melimine or Mel4 with ciprofloxacin was determined after adding equal volumes of each at their corresponding MICs. Wells containing bacteria and MHB and treated with buffer served as negative controls. Following incubation, the media were removed, and wells were then carefully washed two times with HEPES buffer to remove non-adherent cells. Subsequently, biofilms were fixed with 200 μL of 99% *v*/*v* methanol for 15 min and then plates were air dried. Finally, biofilms were stained with 200 μL of 1% *w*/*v* crystal violet dissolved in water for 5 min. Unbound crystal violet was rinsed off with tap water and plates were inverted to air dry. The crystal violet absorbed in biofilms was solubilized in 200 μL glacial acetic acid (33%, *v*/*v*), the released dye was moved to new well and the amount of dye released was determined spectroscopically at OD_600nm._ The degree of biofilm inhibition was determined as a percentage of the biofilm produced by the negative controls (bacteria with no antimicrobials) using the following formulae [62].

(1) $$\begin{array}{c} \%\;\mathrm{biofilm}\;\mathrm{of}\;\mathrm{single}\;\mathrm{or}\;\mathrm{combined}\;\mathrm{antimicrobial}\;\\ =\frac{\left({\mathrm{OD}}_{600\mathrm{nm}}\;\mathrm{of}\;\mathrm{negative}\;\mathrm{control}\right)\;-\;\left({\mathrm{OD}}_{600\mathrm{nm}}\;\mathrm{of}\;\mathrm{individual}\;\mathrm{or}\;\left(\mathrm{combined}\right)\;\mathrm{antimicrobials}\right)}{\left({\mathrm{OD}}_{600\mathrm{nm}}\;\mathrm{of}\;\mathrm{negative}\;\mathrm{control}\right)}\times 100 \end{array}$$

### 4.5. Disruption of Pre-Formed Biofilms by AMPs and Ciprofloxacin Alone or in Combination

Biofilms were formed by adding 100 µL of *S. aureus* ATCC 25923 (1 × 10^6^ CFU/mL) ciprofloxacin-sensitive or resistant cells into round-bottom 96-well microtiter plates containing 100 µL of MHB. Plates were incubated at 37 °C in static condition. After incubation, biofilms were treated with serially diluted peptides or ciprofloxacin or their combination at their corresponding MICs and the plates were incubated for a further 24 h at 37 °C in static condition. Wells containing bacteria and MHB and treated with buffer served as negative controls. Following incubation, the media were removed, and wells were then carefully washed two times with HEPES buffer to remove non-adherent cells and the amount of biofilm was determined as outlined in the previous experiment.

The ability of each antimicrobial to disrupt pre-formed biofilms formed by resistant (30-day ciprofloxacin-passaged) *S. aureus* ATCC 25923 was visualized with confocal laser scanning microscopy (FV 1200, Olympus, Tokyo, Japan). A 24 h pre-formed biofilm on sterile round glass coverslips in polystyrene plates was treated with 200 µL of 4X-MIC of melimine, Mel4 or ciprofloxacin alone or in combination at 37 °C for 24 h. Thereafter, biofilms were stained with Live/Dead BacLight bacterial viability kit (Invitrogen, Eugene, OR, USA) and examined with confocal microscopy. The resulting data were processed using the Image J software version 8 (Bethesda, MD, USA).

### 4.6. Mechanistic Studies

As both AMPs had similar antibiofilm effects against either 1-day or 30-day ciprofloxacin-passaged strains of *S. aureus* ATCC 25923, the 30-day ciprofloxacin-passaged cells were selected to evaluate the mechanism of action of both the AMPs and ciprofloxacin towards bacterial cells in biofilms.

### 4.7. Effect on Cell Membranes

The depolarizing effect on the cell membranes of biofilm-embedded cells was determined as described previously [48].

Briefly, 24 h formed biofilms were washed with 5 mM HEPES (pH 7.2) containing 20 mM glucose and 100 mM KCl at pH 7.2. Then, biofilm cells were loaded with the membrane potential sensitive dye DiSC3 (5) (4 µM; Sigma Aldrich, St Louis, MO, USA)) in HEPES for 1 h in dark. Release of DiSC3 (5) following addition of serially diluted melimine, Mel4 or ciprofloxacin alone or in combination at 1X, 2X and 4X their respective MICs was recorded at regular intervals up to 6 h. DMSO (20%; Merck, Billerica, MA, USA) was used as a positive control to achieve maximum membrane depolarization.

### 4.8. Release of Cellular Contents

The biofilm cells were incubated with serially diluted melimine, Mel4 or ciprofloxacin alone or in combination at 1X, 2X and 4X their corresponding MICs. The supernatants were removed after 3 h and filtered through 0.22 µm pore membranes (Merck, Tullagreen, Ireland). Subsequently, the amount of extracellular of ATP was measured using a bioluminescence kit (Invitrogen, Eugene, OR, USA) according to manufacturer’s instructions. Buffer (HEPES)-treated samples were used as negative controls [47].

Similarly, supernatant was also analyzed for release of nucleic acids (DNA/RNA) [26]. The supernatants were centrifuged at 1300× *g* for 10 min and then filtered through 0.22 µm pore membranes (Merck). The OD_260nm_ of the filtrate was measured, and the results were expressed relative to the initial OD_260nm_ of biofilms taken at 0 min. Furthermore, the presence of nucleic acids in the supernatants was also confirmed with Sytox green (5 µM Invitrogen, Eugene, OR, USA) as final concentration. An increase in fluorescence due to the interaction of Sytox green with nucleic acid was measured spectrophotometrically at an excitation wavelength of 480 nm and an emission wavelength of 523 nm.

### 4.9. Statistical Analysis

All experiments were performed in three independent assays. One-way analysis of variance (ANOVA) with Bonferroni’s corrections for multiple comparisons was used to compare differences between control and antimicrobial-treated cells. The data of cell membrane depolarization were analyzed using two-way ANOVA with Tukey’s test. A probability value of *p* < 0.05 was considered statistically significant.

## 5. Conclusions

In conclusion, *S. aureus* in suspension could not become resistant to melimine or Mel4 following repeated exposure in sub-inhibitory concentrations of these AMPs. Whilst both AMPs inhibited biofilm formation, once *S. aureus* had produced a biofilm, the cells became more resistant to melimine or Mel4, although they could still act against the biofilms at 4X their MICs. Moreover, the combination of the AMPs and ciprofloxacin produced greater effects, possibly as a result of the AMPs damaging the cell membrane of biofilm cells which resulted in increased or facilitated uptake of ciprofloxacin. Future research should be conducted, using, for example, fluorescently labelled ciprofloxacin to examine whether the combination results in greater uptake of ciprofloxacin.

## Figures and Tables

**Table 1:** MIC and MBC values of melimine, Mel4 and ciprofloxacin against *S. aureus*.

Bacterial Strains	Melimine		Mel4		Ciprofloxacin	
	MIC µM (µg·mL^−1^)	MBC µM (µg·mL^−1^)	MIC µM (µg·mL^−1^)	MBC µM (µg·mL^−1^)	MIC µM (µg·mL^−1^)	MBC µM (µg·mL^−1^)
*S. aureus* 31	33.01 (125)	66.02 (250)	106.48 (250)	212.96 (500)	1.50 (0.5)	3.01 (1)
*S. aureus* 38	33.01 (125)	66.02 (250)	106.48 (250)	212.96 (500)	1.50 (0.5)	3.01 (1)
*S. aureus* ATCC 6538	16.50 (62.5)	16.50 (62.5)	53.24 (125)	53.24 (125)	1.50 (0.5)	1.50 (0.5)
*S. aureus* ATCC 25923	33.01 (125)	66.02 (250)	212.96 (500)	212.96 (500)	1.50 (0.5)	3.01 (1)

MBC = minimum bactericidal concentration that kills ≥ 99.99% of bacteria of bacterial population compared to positive control; MIC = minimum inhibitory concentration that kills ≥ 90% of bacterial population when compared to the positive control.

**Table 2:** Effect of antimicrobial peptides and antibiotics at 0.5X MIC in combination against *S. aureus* biofilm.

Antimicrobial Agents	Biofilm Inhibition (%)	Biofilm Eradication (%)
Melimine + Ciprofloxacin	91%	69%
Mel4 + Ciprofloxacin	83%	86%
Citropin1.1 + Minocycline [54]	>99%	ND
Indolicidin + Daptomycin [53]	44%	ND
Nisin + Ciprofloxacin [53]	50%	ND
LL37 + Teicoplanin [20]	ND	>99%
Temporin A +Gentamycin [57]	ND	90%
Indolicidin + Ciprofloxacin [38]	ND	47%
